# Prevalence study and risk factor analysis of selected bacterial, protozoal and viral, including vector-borne, pathogens in cats from Cyprus

**DOI:** 10.1186/s13071-017-2063-2

**Published:** 2017-03-13

**Authors:** Charalampos Attipa, Kostas Papasouliotis, Laia Solano-Gallego, Gad Baneth, Yaarit Nachum-Biala, Elpida Sarvani, Toby G. Knowles, Sena Mengi, David Morris, Chris Helps, Séverine Tasker

**Affiliations:** 1 Molecular Diagnostic Unit, Diagnostic Laboratories, Langford Vets and School of Veterinary Sciences, University of Bristol, Langford, UK; 2 Cyvets Veterinary Center, Paphos, Cyprus; 3 Departament de Medicina i Cirurgia Animals, Facultat de Veterinària, Universitat Autònoma de Barcelona, Barcelona, Spain; 4 Koret School of Veterinary Medicine, Hebrew University, Rehovot, Israel; 5 School of Veterinary Sciences, University of Bristol, Langford, UK; 6 Petcare Veterinary Clinic, Nicosia, Cyprus

**Keywords:** Cyprus, Feline vector-borne pathogens, *Leishmania infantum*, *Bartonella henselae*, *Anaplasma platys*, *Hepatozoon felis*, Haemoplasma, FeLV, FIV

## Abstract

**Background:**

Feline infectious agent studies are lacking in Cyprus. The aims of this study were to determine the prevalence and risk factors for various feline infectious agents, including feline vector-borne pathogens (FVBP), in cats from Cyprus.

**Methods:**

A cross-sectional, descriptive, multicentre study was performed on 174 feline samples [138 owned and 36 shelter-feral, including both healthy (43) and non-healthy (131), cats] from private veterinary clinics from all six districts of Cyprus. Real-time quantitative polymerase chain reaction (qPCR) assays were used to detect *Mycoplasma haemofelis* (Mhf), “*Candidatus* Mycoplasma haemominutum” (CMhm) and “*Candidatus* Mycoplasma turicensis” (CMt). The population was tested for four FVBP including *Bartonella henselae* and *Leishmania* spp. using qPCR, while conventional PCR assays were used to detect *Ehrlichia/Anaplasma* spp. and *Hepatozoon* spp. Serological assays were performed to detect *Leishmania infantum* antibodies, feline leukaemia virus (FeLV) antigen and feline immunodeficiency virus (FIV) antibodies. Statistical analysis was performed to test associations and possible risk factors between variables and infectious agents.

**Results:**

Ninety-six (55.2%) of the 174 cats were PCR-positive for at least one infectious agent. Forty-six cats (26.4%) were haemoplasma positive, including 13 (7.5%) for Mhf, 36 (20.7%) for CMhm and 12 (6.9%) for CMt. Sixty-six cats (37.9%) were positive for *Hepatozoon* spp., while 19 (10.9%) were positive for *B. henselae*, four (2.3%) for *Leishmania* spp. and one (0.6%) for *Ehrlichia/Anaplasma* spp. Sequencing revealed the presence of *Hepatozoon felis*, *L. infantum* and *Anaplasma platys*. Of the 164 cats that underwent retroviral serology, 10 (6.1%) were FeLV-positive and 31 (18.9%) were FIV-positive, while *L. infantum* serology was positive in 7 (4.4%) of the 160 cats tested. Multivariable logistic regression revealed significant associations for various infectious agents including *L. infantum* with each of *Hepatozoon* spp. and CMt infection.

**Conclusions:**

A high prevalence of infectious agents was found in cats from Cyprus with Mhf, CMhm, CMt, *L. infantum*, *B. henselae*, *H. felis*, *A. platys*, FeLV and FIV infections reported for the first time. The significant associations between different pathogens provide a better understanding of similarities in the epidemiology of these pathogens and interactions between them.

**Electronic supplementary material:**

The online version of this article (doi:10.1186/s13071-017-2063-2) contains supplementary material, which is available to authorized users.

## Background

The Republic of Cyprus is an island state located at the crossroads between Europe, Asia and Africa, with the first evidence of cat domestication reported 9,500 years ago [1]. It is the third largest Mediterranean island with a territory of 9,251 km^2^ of which almost half is dominated by mountain ranges. The climate of Cyprus is warmer than the temperate climate typical of some other European Mediterranean countries. This, combined with the geographical location and other factors, favours the maintenance of many arthropod vectors including ticks, fleas, phlebotomine sand flies and mosquitoes [2–6].

While many studies on feline haemoplasmas, feline leukaemia virus (FeLV) and feline immunodeficiency virus (FIV) have been performed worldwide, feline vector-borne pathogens (FVBP) have only been studied relatively recently, and are showing an expanding distribution [7–16]. This illustrates the potential cats have for maintaining and distributing vector-borne pathogens (VBP), some of which are zoonotic.

Vector-borne pathogens have been identified in the government-controlled southern part of Cyprus in various host animal species; *Leishmania infantum*, *Ehrlichia canis*, *Anaplasma platys*, *Hepatozoon canis*, *Babesia vogeli* and *Mycoplasma haemocanis* have been reported in dogs [6, 17], and several rickettsial agents have been reported in goats, sheep, cattle, dogs, mouflon, foxes and hares [2, 3]. Until now, no epidemiological studies have been performed for any infectious agent in cats from Cyprus nor in any small animal species from the non-government-controlled northern part of the country.

The aims of this study were to investigate the presence of several infectious agents, including some FVBP with zoonotic concern, in cats from the whole of Cyprus and to identify risk factors associated with them using multivariable logistic regression. Specifically, we investigated feline haemoplasmas [*Mycoplasma haemofelis* (Mhf), “*Candidatus* Mycoplasma haemominutum” (CMhm) and “*Candidatus* Mycoplasma turicensis” (CMt)], *Bartonella henselae*, *Hepatozoon* spp., *Leishmania* spp. and *Ehrlichia/Anaplasma* spp. using DNA-based detection techniques. Additionally, specific antibodies for FIV and *Leishmania infantum* antigens were determined and antigenaemia was assessed for FeLV.

## Methods

### Animals and samples

From March to September 2014, a total of 176 cats from veterinary clinics in Cyprus were studied. Cats were from urban and rural areas of all six districts of the island; Paphos, Nicosia, Larnaca, Limassol, Famagusta and Kyrenia. Surplus EDTA-blood (0.5–1.0 ml), and when possible serum (0.5–1.0 ml), were collected from cats following written consent from the cat owner or person in charge of the animal shelter. The healthy cat samples comprised pre-anaesthetic screens or samples collected for check-ups (e.g. pre- or post-traveling) whilst the samples from clinically ill animals were taken for diagnostic investigations.

Samples were stored at -20 °C until transported on dry ice to the Diagnostic Laboratories, Langford Vets, University of Bristol, UK, for testing. Data on age, gender (male or female), breed (non-pedigree or pedigree), housing (access to outdoors or indoors only), lifestyle (shelter-feral or owned), district of cat origin in Cyprus (Paphos, Nicosia, Larnaca, Limassol, Famagusta or Kyrenia), habitat (rural or urban), any previous travel history abroad (never travelled abroad or travelled abroad) and health status (non-healthy or healthy, determined by the veterinarian) were registered for each cat. Whenever available, data on the cat’s vaccination status (never vaccinated or vaccinated), use of ectoparasitic prevention (never used or used) and presence of anaemia (haematocrit < 25%), based on in-house complete blood count, were also recorded.

### Polymerase chain reaction (PCR) tests

The DNA was extracted from 100 μl of EDTA blood using a commercial kit (Macherey-Nagel nucleospin blood kit, Düren, Germany) according to the manufacturer's instructions. During extraction nuclease-free water was used as a negative extraction control. The DNA was eluted with 100 μl of elution buffer provided with the kit and stored at -20 °C prior to analysis.

In order to assess the presence of amplifiable DNA, the absence of PCR inhibitors and correct assay setup, all quantitative (q) PCRs were duplexed with an internal amplification control. For the haemoplasma qPCRs, the feline 28S rRNA gene was used and a threshold cycle (Ct) cut-off value of < 30 was used to indicate adequate amplifiable DNA. For the *Leishmania* spp. and *B. henselae* qPCRs, the glyceraldehyde-3-phosphate dehydrogenase gene was used and a Ct value of < 27 was used as a cut-off. Any samples with Ct values greater than or equal to the cut-off values were excluded from the study due to insufficient quantity/quality of DNA. Multiplex qPCR assays, as previously described, were used to detect infection with Mhf, CMhm, CMt [18], *Leishmania* spp. (screening assay) [19] and *B. henselae* [20], and a conventional PCR, as previously described, was used to detect infection with *Ehrlichia/Anaplasma* spp. [21]. Table 1 lists all the primer sequences and products sizes for the PCR assays used. A novel PCR assay was designed and validated (see below) for the detection of *Hepatozoon* spp. For each assay, DNA from known infected cats (or dogs for *Ehrlichia/Anaplasma* spp., *Hepatozoon* spp. and *Leishmania* spp.) and nuclease-free water were used as positive and negative controls, respectively.

**Table 1:** Polymerase chain reaction details for the qPCR/PCR assays used in the study for the testing of infectious agents

Target species (target gene)	PCR primer or probe sequences (5'–3')	Product size (bp)	Reference
*Bartonella henselae* (*alr-gcvP* intergenic spacer)	F: GAGGGAAATGACTCTCTCAGTAAAA	110	[20]^a^
	R: TGAACAGGATGTGGAAGAAGG		
	FAM-CAGCCAAATATACGGGCTATCCATCAA-TAMRA		
*“Candidatus* Mycoplasma haemominutum” (16S rRNA gene)	F: TGATCTATTGTKAAAGGCACTTGCT	135	[18]
	R: TTAGCCTCYGGTGTTCCTCAA		
	FAM-TTCAATGTGTAGCGGTGGAATGCGT-BHQ1		
*“Candidatus* Mycoplasma turicensis” (16S rRNA gene)	F: AGAGGCGAAGGCGAAAACT	138	[18]
	R: ACGTAAGCTACAACGCCGAAA		
	FAM-CGTAAACGATGGGTATTAGATGTCGGGAT-BHQ1		
*Ehrlichia/Anaplasma* spp*.* (16S rRNA gene)	F: GGTACCYACAGAAGAAGTCC	345	[21]
	R: TAGCACTCATCGTTTACAGC		
*Hepatozoon*. spp. (18S rRNA gene)	F: AAACGGCTACCACATNTAAGGA	522	
	R: AATACAAATGCCCCCAACTNT		
*Leishmania* spp. (screening assay) (kinetoplast DNA, kDNA)	F: CGGGTAGGGGCGTTCTG	115	[19]
	R: ATTTTACACCAACCCCCAGTT		
	FAM-TGGGTGCAGAAATCCCGTTCA-BHQ1		
*Leishmania* spp. (confirmatory assay) (kDNA)	F: CCTATTTTACACCAACCCCCAGT	120	[22, 23]
	R: GGGTAGGGGCGTTCTGCGAAA		
*Mycoplasma haemofelis* (16S rRNA gene)	F: GTGCTACAATGGCGAACACA	80	[18]
	R: TCCTATCCGAACTGAGACGAA		
	FAM-TGTGTTGCAAACCAGCGATGGT-BHQ1		

*Abbreviations*: *F* forward primer sequence, *R* reverse primer sequence, *FAM* 6-carboxyfluorescein on the Taqman probe, *BHQ1* black hole quencher 1 on the Taqman probe, *TAMRA* Carboxytetramethylrhodamine on the Taqman probe

^a^The reverse and probe sequences in the original paper are incorrectly labelled; the correct sequences are cited in this table

The DNA from six samples that yielded positive results with the screening *Leishmania* spp. qPCR assay were shipped to the Koret School of Veterinary Medicine, Hebrew University, Rehovot, Israel for confirmatory *Leishmania* spp. qPCR analysis, using a previously described assay [22, 23].

### Novel *Hepatozoon* spp. PCR assay

The PCR assay for *Hepatozoon* spp. was based on the 18S rRNA gene. All available sequences larger than 1,000 bp for *Hepatozoon felis*, *H. canis* and *Hepatozoon americanum* were downloaded from GenBank (https://www.ncbi.nlm.nih.gov/genbank/) and aligned using CLC Sequence Viewer 6.7.1. The 100% consensus sequence was used with Primer3 (http://bioinfo.ut.ee/primer3-0.4.0/primer3) to design primers and MFold (http://unafold.rna.albany.edu/?q=mfold) was used to predict likely secondary structures within the amplicon. The primers, Hep for (5'-AAA CGG CTA CCA CAT NTA AGG A-3') and Hep rev (5'-AAT ACA AAT GCC CCC AAC TNT-3') were chosen, amplifying a PCR product of 504 bp for *H. canis* and *H. felis* and 522 bp for *H. americanum*. Primers were synthesised by Metabion International (Steinkirchen, Germany).

Amplification was performed in a PeqStar 2X thermocycler (Peqlab, Erlangen Germany). A final volume of 25 μl, containing 12.5 μl of 2× GoTaq G2 Master Mix (Promega, Madison, USA), 7 μl of nuclease-free water, 0.5 μl of forward and reverse primer mix at 10 μM each and 5 μl of DNA template, was used. Thermocycler conditions were set at 95 °C for 2 min, followed by 40 cycles of 95 °C for 15 s and 60 °C for 45 s. The DNA of a dog and a cat previously diagnosed with *H. canis* and *H. felis*, respectively, based on positive *Hepatozoon* spp. PCR [24] and 18S rRNA gene sequencing, were used as positive controls. Nuclease-free water was used as a negative control. All amplicons were run on a 2% agarose gel (Appleton Woods, Birmingham, UK), using 1X TAE buffer (Thermo Fisher Scientific, Paisley, UK) and ethidium bromide (Sigma-Aldrich, St. Louis, USA) at a final concentration of 50 ng/ml of gel, at 100 V for 40 min and an image of the gel was captured under ultraviolet light.

Specificity was evaluated using samples known to contain *H. felis*, *H. canis*, *B. canis*, *Babesia rossi*, *E. canis*, *Anaplasma phagocytophilum*, *L. infantum*, *Bartonella clarridgeiae*, Mhf, CMhm, CMt, *M. haemocanis*, “*Candidatus* M. haematoparvum”, *Neospora caninum* and *Toxoplasma gondii* DNA. Any amplicon produced during the validation was purified using the NucleoSpin PCR and Gel Clean-up kit (Macherey-Nagel, Düren, Germany) according to the manufacturer's instructions, quantified with a Qubit™ fluorometer (Thermo Fisher Scientific, Paisley, UK) and submitted for DNA sequencing at DNA Sequencing and Services (College of Life Sciences, University of Dundee, Scotland), in both directions using the same primers as those used for the PCR.

### DNA sequencing

Fourteen of the 66 *Hepatozoon* spp. positive samples (due to financial constraints) from cats living in all 6 districts of Cyprus, and the *Ehrlichia/Anaplasma* spp. positive sample were purified, quantified and submitted for DNA sequencing as described above. All amplicons from the confirmatory *Leishmania* spp. qPCR were also sequenced using the BigDye Terminator v3.1 Cycle Sequencing Kit and an ABI PRISM 3100 Genetic Analyzer (Applied Biosystems, Foster city, USA), at the Center for Genomic Technologies, Hebrew University of Jerusalem, Israel. Forward and reverse DNA sequences were assembled, constructed into consensus sequences and aligned for identification of infecting species according to the closest NCBI BLAST (www.ncbi.nlm.nih.gov/BLAST) [25] match against previously deposited GenBank sequences. *Hepatozoon* spp. (KY215805-KY215818) and *Ehrlichia/Anaplasma* spp. (KY212527) sequences derived from this study were deposited in the GenBank database. The sequences from the confirmatory *Leishmania* spp. qPCR were not deposited in GenBank since these species have already been described in dogs from Cyprus [6].

### FeLV and FIV serology

The PetCheck FeLV Antigen Test and PetCheck FIV Antibody Test (IDEXX Laboratories, Westbrook, Maine, USA) were used for the detection of FeLV antigens and antibodies against FIV in the 164 available cat sera samples, respectively, following the manufacturer’s instructions.

### *Leishmania infantum* serology

Available cat sera from 160 cases were shipped to the Departament de Medicina i Cirurgia Animal, Facultat de Veterinaria, Universitat Autonoma de Barcelona, Spain for *L. infantum* enzyme-linked immunosorbent assay (ELISA) testing using a previously described protocol [26]. A cut-off was established at 32 ELISA units for IgG (mean ± 3 standard deviations). Each sample was quantified as ELISA units (EU) relative to a positive control calibrator cat serum sample, arbitrarily set at 100 EU, which was included on each plate. A negative control cat serum, from a cat known not to be *Leishmania*-infected, was also included on each plate.

### Statistical analysis

Only samples that were positive for both qPCR internal controls using the stipulated Ct cut-offs were included in the statistical analysis carried out using SPSS for Windows (version 22.0; SPSS Inc., Chicago IL, USA).

For statistical analysis, four groups of infectious agents were formed comprising “Any haemoplasma” (positivity in at least one of the following qPCRs; Mhf, CMhm and CMt), “*L. infantum* infection” (positive DNA sequencing for *L. infantum* following confirmatory qPCR and/or positive *L. infantum* ELISA), “Retroviral serology” (positive for FeLV and/or FIV serology) and “FVBP” [positive for at least one of the PCRs for *B. henselae*, *Ehrlichia/Anaplasma* spp. and/or *Hepatozoon* spp., and/or *L. infantum* infection (i.e. positive DNA sequencing for *L. infantum* following confirmatory qPCR and/or positive *L. infantum* ELISA)].

The Kolmogorov-Smirnov test was used to assess for normality of distribution of the continuous variable age. Mann-Whitney U-tests were then used to evaluate for differences between non-normally distributed variable of age across infectious agent group(s). Initial analyses using Chi-square test was performed to evaluate any associations between the 19 categorical variables across individual infectious agent group(s). Multivariable logistic regression was used to test for possible risk factors associated with infection. Independent variables that yielded *P-*values of < 0.2 in a univariable analysis were then tested in a multivariable logistic regression analysis. Backward selection was used primarily, and once a final model was constructed all the previously excluded variables were then individually retested and, if then significant, were included within the final model. Within the final multivariable models a *P-*value ≤ 0.05 was considered statistically significant for inclusion, and the *P-*values with odds ratio (OR) and 95% confidence interval (CI) are reported.

## Results

Of the 176 DNA samples analysed, two were excluded due to failure of one or more of the internal amplification control qPCRs, hence 174 samples were used in the study and subsequent statistical analyses. The age of these 174 cats ranged from 0.4 to 22.0 years (median 5.6 years, interquartile range 8 years) and only 15 (8.6%) were pedigree including six Ragdolls, six Persians, two Siamese and one Russian Blue. Tables 2 and 3 show descriptive statistics as well as data on the prevalence of infectious agents among the population studied.

**Table 2:** Comparison of prevalence of infectious agents detected by PCR in cats from Cyprus per categorical variable

Variable/category	No. of cats (%)	No. of PCR positive cats (%)						
		Mhf	CMhm	CMt	Any hp	*Hepatozoon* spp.	*B. henselae*	*L. infantum*
Gender	174							
Male	96 (55.2)	7 (7.3)	21 (21.9)	6 (6.3)	25 (26.0)	34 (35.4)	10 (10.4)	2 (2.1)
Female	78 (44.8)	6 (7.7)	15 (19.2)	6 (7.7)	21 (26.9)	32 (41.0)	9 (11.5)	2 (2.6)
Breed	174							
Non-Pedigree	159 (91.4)	13 (8.2)	35 (22.0)	12 (7.6)	45 (28.3)	65 (40.9)	19 (12.0)	4 (3.8)
Pedigree	15 (8.6)	0 (0)	1 (6.7)	0 (0)	1 (6.7)	1 (6.7)	0 (0)	0 (0)
Housing	174							
Access to outdoors	134 (77.0)	13 (9.7)	35 (26.1)	10 (7.5)	44 (32.8)	55 (41.1)	17 (12.7)	4 (3.0)
Indoors only	40 (23.0)	0 (0)	1 (2.5)	2 (5.0)	2 (5.0)	11 (27.5)	2 (5.0)	0 (0)
Lifestyle	174							
Shelter-feral	36 (20.7)	5 (13.9)	24 (66.6)	6 (16.7)	14 (38.9)	20 (55.6)	6 (16.7)	1 (2.8)
Owned	138 (79.3)	8 (5.8)	12 (8.7)	6 (4.3)	32 (23.2)	46 (33.3)	13 (9.4)	3 (2.2)
District	174							
Paphos	59 (33.9)	4 (6.8)	14 (23.7)	4 (6.8)	16 (27.1)	23 (39.0)	10 (17.0)	4 (6.8)
Nicosia	51 (29.4)	3 (5.9)	7 (13.7)	2 (3.9)	10 (19.6)	21 (41.2)	4 (7.8)	0 (0)
Larnaca	28 (16.1)	2 (7.1)	5 (17.9)	1 (3.6)	6 (21.4)	8 (28.6)	2 (7.1)	0 (0)
Limassol	22 (12.6)	2 (9.1)	8 (36.4)	3 (13.6)	9 (40.9)	7 (31.8)	3 (13.6)	0 (0)
Famagousta	7 (4.0)	0 (0)	0 (0)	0 (0)	0 (0)	4 (57.1)	0 (0)	0 (0)
Kyrenia	7 (4.0)	2 (28.6)	2 (28.6)	2 (28.6)	5 (71.4)	3 (42.9)	0 (0)	0 (0)
Habitat	174							
Rural	65 (37.4)	7 (10.8)	17 (26.2)	6 (9.2)	21 (32.3)	33 (50.8)	8 (12.3)	2 (3.1)
Urban	109 (62.6)	6 (5.5)	19 (17.4)	6 (5.5)	25 (22.9)	33 (30.3)	11 (10.1)	2 (1.8)
Travel history	174							
Never travelled abroad	159 (91.4)	13 (8.2)	32 (20.1)	11 (6.9)	42 (26.4)	65 (40.9)	17 (10.7)	4 (2.5)
Travelled abroad	15 (8.6)	0 (0)	4 (26.7)	1 (6.7)	4 (26.7)	1 (6.7)	2 (13.3)	0 (0)
Health status	174							
Non-healthy	131 (75.3)	12 (9.2)	33 (25.2)	9 (6.9)	41 (31.3)	58 (44.3)	14 (10.7)	4 (3.1)
Healthy	43 (24.7)	1 (2.3)	3 (6.9)	3 (7.0)	5 (11.6)	8 (18.6)	5 (11.6)	0 (0)
Vaccination status	165							
Never vaccinated	47 (28.5)	8 (17.0)	9 (19.2)	7 (14.9)	14 (29.8)	25 (53.2)	4 (8.5)	1 (2.1)
Vaccinated	118 (71.5)	5 (4.2)	23 (19.5)	5 (4.2)	28 (23.7)	36 (30.5)	15 (12.7)	3 (2.5)
Ectoparasitic prevention status	165							
Never used	62 (37.6)	8 (12.9)	15 (24.2)	7 (11.3)	21 (33.9)	31 (50.0)	9 (14.5)	2 (3.2)
Used	103 (62.4)	5 (4.9)	17 (16.5)	5 (4.9)	21 (20.4)	30 (29.1)	10 (9.7)	2 (1.9)
Anaemia	132							
Anaemic	29 (22.0)	3 (10.4)	9 (31.0)	3 (10.3)	9 (31.0)	13 (44.8)	3 (10.4)	1 (3.5)
Non-anaemic	103 (78.0)	6 (5.8)	18 (17.5)	5 (4.9)	23 (22.3)	33 (32.0)	16 (15.5)	3 (2.9)
FeLV	164							
Positive	10 (6.1)	1 (10.0)	3 (30.0)	2 (20.0)	3 (30.0)	5 (50.0)	0 (0)	1 (10.0)
Negative	154 (93.9)	10 (6.5)	30 (19.5)	9 (5.8)	39 (25.3)	59 (38.3)	18 (11.7)	3 (2.0)
FIV	164							
Positive	31 (18.9)	4 (12.9)	17 (54.8)	7 (22.6)	19 (61.3)	17 (54.8)	5 (16.1)	1 (3.2)
Negative	133 (81.1)	7 (5.3)	16 (12.0)	4 (3.0)	23 (17.3)	47 (35.3)	13 (9.8)	3 (2.3)
Total	174	13 (7.5)	36 (20.7)	12 (6.9)	46 (26.4)	66 (37.9)	19 (10.9)	4 (2.3)

*Abbreviations*: *Mhf Mycoplasma haemofelis*, *CMhm* “*Candidatus* Mycoplasma haemominutum”, *CMt* “*Candidatus* Mycoplasma turicensis”, *Any hp* positivity in at least one of the following haemoplasma PCR; Mhf, CMhm and CMt, *B. henselae Bartonella henselae*, *L. infantum Leishmania infantum* confirmed by DNA sequencing following confirmatory quantitative PCR, *FeLV* feline leukaemia virus, *FIV* feline immunodeficiency virus

*Note*: Only one cat was positive for *Ehrlichia/Anaplasma* spp. PCR and information regarding this case is reported in the results section of the main text

**Table 3:** Comparison of prevalence of infectious agents in cats detected by serology from Cyprus per categorical variable

Variable/category	No. of cats (%)	No. of serology positive cats (%)		No. of cats (%)	No. of serology positive cats for *L. infantum* (%)
		FeLV	FIV		
Gender	164			160	
Male	88 (53.7)	7 (8.0)	18 (20.5)	86 (53.8)	3 (3.5)
Female	76 (46.3)	3 (4.0)	13 (17.1)	74 (46.2)	4 (5.4)
Breed	164			160	
Non-Pedigree	149 (90.9)	10 (6.7)	31 (20.8)	147 (91.9)	7 (4.8)
Pedigree	15 (9.2)	0 (0)	0 (0)	13 (8.1)	0 (0)
Housing	164			160	
Access to outdoors	125 (76.2)	5 (4.0)	29 (23.2)	124 (77.5)	7 (5.7)
Indoors only	39 (23.8)	5 (12.8)	2 (5.1)	36 (22.5)	0 (0)
Lifestyle	164			160	
Shelter-feral	33 (20.1)	1 (3.0)	14 (42.4)	33 (20.6)	3 (9.1)
Owned	131 (79.9)	9 (6.9)	17 (13.0)	127 (79.4)	4 (3.2)
District	164			160	
Paphos	57 (34.8)	1 (1.8)	13 (22.8)	56 (35.0)	1 (1.8)
Nicosia	48 (29.3)	1 (2.1)	8 (16.7)	44 (27.5)	3 (6.8)
Larnaca	25 (15.2)	3 (12.0)	3 (12.0)	26 (16.3)	0 (0)
Limassol	21 (12.8)	2 (9.5)	5 (23.8)	21 (13.1)	3 (14.3)
Famagousta	7 (4.3)	1 (14.3)	0 (0)	7 (4.4)	0 (0)
Kyrenia	6 (3.7)	2 (33.3)	2 (33.3)	6 (3.7)	0 (0)
Habitat	164			160	
Rural	60 (36.6)	3 (5.0)	13 (21.7)	62 (38.8)	3 (4.8)
Urban	104 (63.4)	7 (6.7)	18 (17.3)	98 (61.2)	4 (4.1)
Travel history	164			160	
Never travelled abroad	149 (90.9)	10 (6.7)	27 (18.1)	145 (90.6)	7 (4.8)
Travelled abroad	15 (9.2)	0 (0)	4 (26.7)	15 (9.4)	0 (0)
Health status	164			160	
Non-healthy	123 (75.0)	10 (8.1)	29 (23.6)	117 (73.1)	6 (5.1)
Healthy	41 (25.0)	0 (0)	2 (4.9)	43 (26.9)	1 (2.3)
Vaccination status	156			151	
Never vaccinated	43 (27.6)	4 (9.3)	8 (18.6)	43 (28.5)	4 (9.3)
Vaccinated	113 (72.4)	6 (5.3)	20 (17.7)	108 (71.5)	2 (1.9)
Ectoparasitic prevention status	156			151	
Never used	57 (36.5)	2 (3.5)	15 (26.3)	58 (38.4)	4 (6.9)
Used	99 (63.5)	8 (8.1)	13 (13.1)	93 (61.6)	2 (2.2)
Anaemia	128			120	
Anaemic	28 (21.9)	4 (14.3)	10 (35.7)	28 (23.3)	2 (7.1)
Non-anaemic	100 (78.1)	3 (3.0)	15 (15.0)	92 (76.7)	1 (1.1)
Total	164	10 (6.1)	31 (18.9)	160	7 (4.4)

*Abbreviations*: *FeLV* feline leukaemia virus, *FIV* feline immunodeficiency virus

Specificity testing for the novel PCR assay for *Hepatozoon* spp. against *B. canis*, *Babesia rossi*, *E. canis*, *A. phagocytophilum*, *L. infantum*, *B. clarridgeiae*, Mhf, CMhm, CMt, *M. haemocanis, “Ca.* M. haematoparvum”, *N. caninum* and *T. gondii* DNA found no evidence of cross-reactivity. Analytical sensitivity of the assay was assessed as follows. An amplicon from a known *H. canis* positive sample was quantified using a Qubit™ fluorometer (Invitrogen™) and gave 13.2 ng/μl. A 10-fold serial dilution was made from 10^-8^ to 10^-12^, and each dilution was amplified in triplicate using the same conditions as described in the methods. Diluting the amplicon to 10^-10^ gave a 3 out of 3 success rate for detection and 10^-11^ a 2 out of 3 success rate; none of the triplicates at the 10^-12^ dilution gave a positive result. Using the amplicon length of 504 bp and concentration of 13.2 ng/μl, the theoretical limit of detection was calculated as being between 1.2 and 12 copies per PCR. Sequencing of the amplicons derived using the *H. canis* and *H. felis* known positive control DNA samples were found to match the expected *H. canis* or *H. felis* sequences.

Ninety-six (55.2%) of the 174 cats were PCR-positive for at least one infectious agent, 79 (45.4%) were positive to at least one FVBP while 17 (9.8%) were positive for two FVBP (Table 4). Forty-six cats (26.4%) were positive for haemoplasmas, including 13 (7.5%) for Mhf, 36 (20.7%) for CMhm and 12 (6.9%) for CMt (Table 2). Sixty-six cats (37.9%) were positive for *Hepatozoon* spp., while nineteen (10.9%) were positive for *B. henselae*. One cat (0.6%) was PCR positive for *Ehrlichia/Anaplasma* spp. This was a 19-year-old, neutered female, domestic shorthair cat from the Paphos area (rural) that was presented for monitoring of chronic kidney disease. The cat had lived in Greece for 12 years, was fully vaccinated, with access to the outdoors and was treated with a preventative ectoparasiticide. No abnormalities or *A. platys* morulae were found on haematological analysis and blood smear examination, and the cat was PCR positive only for *Hepatozoon* spp. and negative for the other infectious agents screened for in the study. Using the *Leishmania* spp. confirmatory qPCR assay, DNA was detected in 4 (2.3%) of the 174 cats and *L. infantum* serology was positive in 7 of the 160 cats tested (4.4%). Only one cat was positive by both *Leishmania* spp. confirmatory qPCR assay and serology, and additionally had cutaneous lesions caused by *Leishmania* infection reported by the veterinarian. Of the 164 cats that underwent retroviral serology, 10 (6.1%) were FeLV, and 31 (18.9%) were FIV, positive (Table 3).

**Table 4:** Prevalence of single infections and co-infections with feline vector-borne pathogens including *Bartonella henselae*, *Ehrlichia/Anaplasma* spp. and *Hepatozoon* spp. determined by PCR, as well as *Leishmania infantum* infection, among 174 cats from Cyprus

Infectious agent(s)	Positive cats	
	No.	%
Single infections	62	35.7
*B. henselae*	11	6.3
*Hepatozoon* spp.	49	28.2
*L. infantum* infection^a^	2	1.2
Co-infections	17	9.8
*Ehrlichia/Anaplasma* spp. and *Hepatozoon* spp.	1	0.6
*B. henselae* and *Hepatozoon* spp.	8	4.6
*L. infantum* infection^a^ positive and *Hepatozoon* spp.	8	4.6
Total	79	45.5

^a^Positive *L. infantum* infection status defined as cats that had positive DNA sequencing for *L. infantum* following confirmatory qPCR and/or positive *L. infantum* ELISA

Out of the 66 samples that were positive for *Hepatozoon* spp., 14 amplicons (accession numbers KY215805 to KY215818) were sequenced and yielded 96–100% similarity to an existing partial 18S rRNA gene for *H. felis* (KC138534) over 504 bp. The cat that was positive on the generic *Ehrlichia/Anaplasma* spp. PCR yielded an amplicon (KY212527) that had 99% similarity to a partial 16S rRNA gene sequence of *A. platys* (KY114935) over 225 bp. The four amplicons of the positive confirmatory *Leishmania* spp. qPCR (Additional file 1) had 93–98% similarity to kinetoplast DNA from an existing GenBank sequence for *L. infantum* (Z35292) over 122 bp.

Univariable analysis showed that many variables had a trend toward significance (*P* < 0.2) for association with the presence of individual, or groups of infectious agent(s) (Table 5, Table 6, Additional file 2: Table S1, Additional file 3: Table S2), and these were entered into the multivariable logistic regression analysis, together with variables having significant associations (*P* ≤ 0.05).

**Table 5:** *P-*values derived from univariable analysis for variables in relation to infectious agent or group of infectious agents’ positivity. *P*-values < 0.2 but > 0.05 are shown in italics. Significant *P*-values ≤ 0.05 are shown in bold

Variable	Mhf PCR	CMhm PCR	CMt PCR	Any hp PCR	*Bh* PCR	*Li* PCR	*Li* serology	*Li* infection	FeLV serology	FIV serology	Retroviral serology	*Hepatozoon* spp. PCR	FVBP
Age	0.934	**0.014**	0.584	*0.055*	*0.152*	0.712	0.849	0.560	**0.023**	0.288	0.760	0.937	0.530
Gender Male/Female (Ref.)	0.920	0.668	0.709	0.896	0.813	0.833	0.854	0.682	0.202	0.585	*0.173*	0.448	0.420
Breed Non-Pedigree/Pedigree (Ref.)	0.250	*0.161*	0.270	*0.069*	*0.156*	0.534	0.424	0.331	0.270	*0.051*	**0.020**	**0.009**	**0.020**
Housing Access to outdoors/Indoors only (Ref.)	**0.041**	**0.001**	0.590	**0.001**	*0.171*	0.269	*0.148*	*0.078*	*0.075*	**0.015**	0.263	*0.121*	*0.062*
Lifestyle Shelter-feral/Owned (Ref.)	*0.086*	**0.026**	**0.007**	**0.042**	*0.187*	0.597	*0.146*	*0.118*	0.335	**0.001**	**0.012**	**0.009**	**0.021**
Habitat Rural/Urban (Ref.)	0.201	*0.169*	0.348	*0.175*	0.650	0.515	0.799	0.933	0.526	0.474	0.946	**0.007**	**0.003**
District	0.370	*0.195*	*0.136*	**0.017**	0.416	0.205	*0.140*	0.414	*0.105*	0.533	0.341	0.728	0.843
Travel history Never travelled abroad/Travelled abroad (Ref.)	0.233	0.655	0.915	0.891	0.832	0.534	0.371	0.294	0.262	0.503	0.972	**0.028**	**0.025**
Health status Non-healthy/Healthy (Ref.)	*0.139*	**0.011**	0.981	**0.011**	0.864	0.246	0.453	0.214	**0.049**	**0.010**	**0.003**	**0.003**	**0.008**
Vaccination status Never vaccinated/Vaccinated (Ref.)	**0.006**	0.960	**0.017**	0.420	0.445	0.876	**0.036**	*0.063*	0.514	0.943	0.861	**0.006**	**0.006**
Ectoparasitic prevention status Never used/Used (Ref.)	0.634	0.226	*0.123*	0.054	0.349	0.603	*0.149*	*0.072*	*0.186*	**0.044**	0.429	**0.007**	**0.008**
Anaemia Anaemic/Non-anaemic (Ref.)	0.344	*0.110*	0.274	0.334	0.482	0.882	0.087	*0.133*	**0.006**	**0.025**	**0.002**	0.202	0.876

*Abbreviations*: *Mhf Mycoplasma haemofelis*, *CMhm “Candidatus* Mycoplasma haemominutum”, *CMt* “*Candidatus* Mycoplasma turicensis”, *Any hp* positivity in at least one of the following haemoplasma PCRs; Mhf, CMhm and CMt, *Bh Bartonella henselae*, *Li Leishmania infantum* confirmed by DNA sequencing following confirmatory quantitative PCR, *Li infection* positive DNA sequencing for *L. infantum* following confirmatory qPCR and/or positive *L. infantum* ELISA, *FeLV* feline leukaemia virus, *FIV* feline immunodeficiency virus, *Retroviral serology* positive for FeLV and/or FIV serology, *FVBP* positive for at least one of the PCRs for *B. henselae*, *Ehrlichia/Anaplasma* spp. and/or *Hepatozoon* spp., and/or *L. infantum* infection (i.e. positive DNA sequencing for *L. infantum* following confirmatory qPCR and/or positive *L. infantum* ELISA), *Ref.* reference category

*Note*: The *P-*values, *χ* ^2^ and degrees of freedom from Chi-square analysis are reported in the Additional file 2. Table S1. The *P*-and *Z*-values derived from Mann-Whitney U-tests are reported in the Additional file 3. Table S2

**Table 6:** *P-*values derived from Chi-square analysis for variables in relation to infectious agent or group of infectious agents’ positivity. *P*-values < 0.2 but > 0.05 are shown in italics. Significant *P*-values ≤ 0.05 are shown in bold

Variable	Mhf PCR	CMhm PCR	CMt PCR	Any hp PCR	*Bh* PCR	*Li* PCR	*Li* serology	*Li* infection	FeLV serology	FIV serology	Retroviral serology	*Hepatozoon* spp. PCR	FVBP
Mhf PCR status Positive/Negative (Ref.)	na	na	na	na	0.596	*0.177*	0.369	*0.064*	0.682	*0.129*	0.338	0.219	0.073
CMhm PCR status Positive/Negative (Ref.)	na	na	na	na	0.962	*0.143*	*0.137*	0.201	0.421	**0.001**	**0.001**	*0.094*	*0.169*
CMt PCR status Positive/Negative (Ref.)	na	na	na	na	0.768	*0.148*	**0.013**	**0.001**	0.744	**0.001**	**0.002**	**0.006**	**0.001**
Any hp PCR status Positive/Negative (Ref.)	na	na	na	na	0.990	0.280	0.510	**0.044**	0.743	**0.001**	**0.001**	*0.107*	**0.035**
*Li* infection status Positive/Negative (Ref.)	*0.064*	0.201	**0.001**	**0.044**	0.231	na	na	na	0.770	*0.152*	0.216	**0.001**	na
*Bh* PCR status Positive/Negative (Ref.)	0.596	0.962	0.768	0.990	na	0.479	0.367	0.231	0.252	0.315	0.732	0.691	na
Retroviral serology status Positive/Negative (Ref.)	0.338	**0.001**	**0.002**	**0.001**	0.732	0.977	0.234	0.216	na	na	na	**0.045**	0.202
*Hepatozoon* spp. PCR status Positive/Negative (Ref.)	0.219	*0.094*	**0.006**	*0.107*	0.691	**0.010**	**0.010**	**0.001**	0.461	**0.048**	**0.045**	na	na

*Abbreviations*: *Mhf Mycoplasma haemofelis*, *CMhm “Candidatus* Mycoplasma haemominutum”, *CMt* “*Candidatus* Mycoplasma turicensis”, *Any hp* positivity in at least one of the following haemoplasma PCRs; Mhf, CMhm and CMt, *Bh Bartonella henselae*, *Li Leishmania infantum* confirmed by DNA sequencing following confirmatory quantitative PCR, *Li infection* positive DNA sequencing for *L. infantum* following confirmatory qPCR and/or positive *L. infantum* ELISA, *FeLV* feline leukaemia virus, *FIV* feline immunodeficiency virus, *Retroviral serology* positive for FeLV and/or FIV serology, *FVBP* positive for at least one of the PCRs for *B. henselae*, *Ehrlichia/Anaplasma* spp. and/or *Hepatozoon* spp., and/or *L. infantum* infection (i.e. positive DNA sequencing for *L. infantum* following confirmatory qPCR and/or positive *L. infantum* ELISA), *Ref.* reference category, *na* not applicable

*Note*: The *P-*values, *χ* ^2^ and degrees of freedom from Chi-square analysis are reported in the Additional file 2: Table S1

Thirteen sets of multivariable logistic regression, one for each infectious agent or group of infectious agents, were constructed using the independent variables that showed at least a trend towards significance (*P* < 0.2) in the univariable analysis. Eight multivariable models yielded significant associations (*P* ≤ 0.05) (Table 7). No multivariable models yielded significant associations for Mhf, *B. henselae*, *L. infantum* PCR, *L. infantum* serology or FeLV.

**Table 7:** Variables for the positivity of infectious agents or groups of infectious agents in cats in Cyprus: multivariable logistic regression models

	Odds ratio (95% CI)	*P-*value
1. CMhm PCR positive		
Retroviral serology status		
Positive	5.8 (2.4–14.0)	0.001
Negative	Ref.	
Age	1.1 (1.1–1.2)	0.017
Lifestyle		
Shelter-feral	2.8 (1.1–7.4)	0.043
Owned	Ref.	
2. CMt PCR positive		
* L. infantum* infection status		
Positive	7.3 (1.4–37.5)	0.018
Negative	Ref.	
Retroviral serology status		
Positive	5.0 (1.3–219.7)	0.021
Negative	Ref.	
3. Any haemoplasma PCR positive		
Retroviral serology status		
Positive	4.6 (2.1–10.4)	0.001
Negative	Ref.	
Housing		
Access to outdoors	8.7 (1.9–39.1)	0.005
Indoors only	Ref.	
4. *L. infantum* infection positive		
* Hepatozoon* spp. PCR status		
Positive	13.5 (1.6-111.1)	0.016
Negative	Ref.	
CMt PCR status		
Positive	5.6 (1.1–29.1)	0.041
Negative	Ref.	
5. FIV serology positive		
Any haemoplasma PCR status		
Positive	6.6 (2.7–15.9)	0.001
Negative	Ref.	
Lifestyle		
Shelter-feral	4.0 (1.6–10.2)	0.004
Owned	Ref.	
6. Retroviral serology positive		
Any haemoplasma PCR status		
Positive	5.3 (2.1–13.4)	0.001
Negative	Ref.	
Anaemia		
Anaemic	3.6 (1.4–9.5)	0.008
Non-anaemic	Ref.	
7. *Hepatozoon* spp. PCR positive		
Health status		
Non-healthy	3.2 (1.3–7.8)	0.010
Healthy	Ref.	
* L. infantum* infection status		
Positive	12.0 (1.4–106.0)	0.025
Negative	Ref.	
Vaccination status		
Never vaccinated	2.2 (1.1–4.7)	0.048
Vaccinated	Ref.	
8. FVBP positive		
Habitat status		
Rural	2.6 (1.3–5.2)	0.006
Urban	Ref.	
CMt PCR status		
Positive	22.5 (2.3–221.2)	0.008
Negative	Ref.	
Health status		
Non-healthy	2.4 (1.1–5.4)	0.042
Healthy	Ref.	
Travel history		
Never travelled abroad	4.3 (1.1–18.0)	0.045
Travelled abroad	Ref.	

*Abbreviations*: *CI* confidence interval, *Ref.* reference category, *CMhm* “*Candidatus* Mycoplasma haemominutum”, *CMt* “*Candidatus* Mycoplasma turicensis”, *L. infantum infection* positive DNA sequencing for *Leishmania infantum* following confirmatory qPCR and/or positive *L. infantum* ELISA, *Any haemoplasma* positivity in at least one of the following haemoplasma PCRs; Mhf, CMhm and CMt, *FIV* feline immunodeficiency virus, *Retroviral serology* positive for FeLV and/or FIV serology, *FVBP* positive for at least one of the PCRs for *B. henselae*, *Ehrlichia/Anaplasma* spp. and/or *Hepatozoon* spp., and/or *L. infantum* infection (i.e. positive DNA sequencing for *L. infantum* following confirmatory qPCR and/or positive *L. infantum* ELISA)

## Discussion

This is the first large-scale study to provide an overview of infectious agents in cats from Cyprus. Feline haemoplasmas, *B. henselae*, *Hepatozoon* spp. (including *H. felis*), *L. infantum* and *A. platys* were detected by PCR (with or without sequencing), while serology revealed infections with FeLV, FIV and *L. infantum* in the feline population of this island. Additionally, significant associations were identified between infectious agents and independent, risk factor variables using multivariable logistic regression, providing a better understanding of the epidemiology and possible risk factors for these infectious agents.

Over the last few decades, feline hepatozoonosis has been increasingly reported worldwide with prevalences being frequently low, but ranging up to 36% depending on geographical location and lifestyle of cats [11, 13, 15, 27–30]. The exact vectors and routes of transmission of feline hepatozoonoses are not known [27], but vectorial transmission likely plays a key role as for other species of *Hepatozoon* in different vertebrate species such as dogs [31]. The results of the present study demonstrate the utility of the novel *Hepatozoon* spp. PCR assay for the detection of *H. canis* and *H. felis* and the absence of cross-reaction with a range of other pathogens. In this study, we found a prevalence of 37.9% for *Hepatozoon* spp. infection in cats, with an even higher prevalence of 55.5% in shelter-feral cats. Amplicon sequencing revealed the presence of *H. felis* only, but we cannot rule out the possibility of some cats being infected with *H. canis* since not all positive PCR products were sequenced due to financial constraints, and *H. canis* has been previously reported to infect cats [27] and has been described in Cyprus [17]. Univariable statistical analysis revealed ten variables associated with *Hepatozoon* spp. infection from which three (non-healthy, *L. infantum* infection positive status and never vaccinated) remained statistically significant in the multivariable logistic regression model. To our knowledge, this is the first time that associations have been found using multivariable logistic regression for feline hepatozoonosis. The association (OR = 3.2, 95% CI: 1.3–7.8, *P* = 0.010) between *Hepatozoon* spp. infection and health status, with non-healthy cats being three times more likely to be *Hepatozoon* spp. infected compared to healthy cats, is interesting since feline hepatozoonosis has been described as being predominantly a sub-clinical infection [27]. This association does not necessarily mean that the cause of the cats’ ill-health was hepatozoonosis, especially since the cats were often co-infected with other pathogens; therefore further studies are needed to identify the clinical implications of hepatozoonosis in cats. Cats with a positive *L. infantum* infection status were 12 times more likely to be infected with *Hepatozoon* spp. (OR = 12.0, 95% CI: 1.4–106.0, *P* = 0.025) compared to cats with *L. infantum* infection negative status. This co-infection is commonly reported in dogs with *H. canis* [32], and it is the first time that such association has been reported in cats. Co-infection with these two protozoans might lead to higher level of circulating parasites due to impaired response of the host immune system [33]. The reason for the significant association between *Hepatozoon* spp. infection and negative vaccination status is unknown, but could be due to an association with an overall lack of preventative health care.

Similar to dogs, *L. infantum* infection in cats is most likely transmitted by phlebotomine sand flies and is currently an emerging zoonotic infectious disease [34]. The current study’s findings of a *L. infantum* PCR-based prevalence of 2.3% (confirmed by DNA sequencing following confirmatory quantitative PCR), *L. infantum* seroprevalence of 4.4% and a combined infection (i.e. positive DNA sequencing for *L. infantum* following confirmatory qPCR and/or positive *L. infantum* ELISA) prevalence of 5.8%, are similar to those reported in other Mediterranean countries [15, 26, 35, 36], although lower than the 14.9% seroprevalence in dogs from Cyprus [6]. Only one sick cat, which had cutaneous lesions caused by *Leishmania* infection, was positive by both serology and PCR with confirmed *L. infantum* on sequencing. Sequencing showed *L. infantum* in another three cats, and this agreed with a previous study in Cyprus on dogs, where also only *L. infantum* was found [6]. The variables of male gender, adult age, rural habitat [37], outdoor lifestyle [34] and retroviral positivity [26], which are all previously reported risk factors for leishmaniosis in cats, were not found to be significant in this study. However, significant associations between *L. infantum* infection status and infection with *Hepatozoon* spp. (OR = 13.5, 95% CI: 1.6–111.1, *P* = 0.016) and CMt (OR = 5.6, 95% CI: 1.1–29.1, *P* = 0.041) were found by multivariable logistic regression. Possible causes of these associations may reflect pathogen-facilitation or phenotypic traits (e.g. aggressiveness) that were not recorded during the study [38].

The prevalence of haemoplasma infection in cats in this study was similar to those reported in other European countries [10, 39–45], with CMhm infection being most common, followed by Mhf and CMt. Multivariable logistic regression analyses (Table 7) showed significant associations between positive retroviral status and each of CMhm (OR = 5.8, 95% CI: 2.4–14.0, *P* =0.001), CMt (OR = 5.0, 95% CI: 1.3–219.7, *P* = 0.021) and overall haemoplasma infection (OR = 4.6, 95% CI: 2.1–10.4, *P* = 0.001). This supports the previous reports that retroviral infections, especially FIV, are risk factors for haemoplasma infection [43, 46]. Consistent with previous studies [40, 45–47], our study also identified additional risk factors including age (OR = 1.1, 95% CI: 1.1–1.2, *P* = 0.017) and being a shelter-feral cat (OR = 2.8, 95% CI: 1.1–7.4, *P* = 0.043) for CMhm infection, and access to outdoors (OR = 8.7, 95% CI: 1.9–39.1, *P* =0.005) for infection with any haemoplasma species. Interestingly, this is the first time *L. infantum* infection in cats (OR = 7.3, 95% CI: 1.4–37.5, *P* = 0.018) has been associated with CMt infection, with *Leishmania* infected cats being seven times more likely to be CMt-positive.

Molecular investigation detected *B. henselae* in 10.9% of the cats in this study, which is amongst the highest prevalence of infection reported in Europe [9–11, 15, 16]. A recent study from southern Italy [9] reported a 21.4% PCR prevalence of *B. henselae* in outdoor cats that had at least one ectoparasite (tick or flea) present on examination. In the current study both indoor and outdoor cats were included, but ectoparasite presence was not assessed. Despite being a zoonotic infection, this is the first time that *B. henselae* has been detected in Cyprus, although 10.5% of rats in Cyprus have been shown to be seropositive for *B. henselae* [48]. Other *Bartonella* species could possibly exist in Cyprus, but were not investigated, thus further studies are needed.

In our study, 18.9 and 6.1% of the cats were seropositive for FIV and FeLV, respectively, findings which are similar to those reported in previous studies [49–53]. Multivariable logistic regression revealed significant associations between FIV and haemoplasma infection (OR = 6.6, 95% CI: 2.7–15.9, *P* = 0.001) and FIV infection and shelter-feral cats (OR = 4.0, 95% CI: 1.6–10.2, *P* = 0.004). In addition, overall retroviral infection was associated with haemoplasma infection (OR = 5.3, 95% CI: 2.1–13.4, *P* = 0.001) and anaemia (OR = 3.6, 95% CI: 1.4–9.5, *P* = 0.008). To our knowledge, this is the first time multivariable logistic regression has documented an association of seropositivity with shelter-feral cats, and retroviral seropositivity with anaemia.

*Anaplasma platys* is considered a VBP, that is widespread in dogs from the Mediterranean basin and has also been reported in dogs, sheep and goats from Cyprus [17, 54, 55]. There are sporadic reports of this canine pathogen in cats from North America and Brazil [7, 56, 57], and recently *A. platys-*like strains were identified in cats from Sardinia, Italy [58]. In this study, we report a case of presumptive *A. platys* infection in a cat from Cyprus based on partial sequencing of 16S rRNA gene. However, further investigation with additional phylogeny and amplification of multiple and longer genes are needed, in order to definitively prove the identity of this pathogen. In this case, as well as in the previous feline case reports [56, 57], the pathogenic role of *A. platys* in cats is not clear.

The overall prevalence of FVBP in this study is higher than those reported in studies performed in other southern European countries [9, 11, 13]. Logistic regression analyses showed significant associations between FVBP infection and rural habitat, as well as never having travelled abroad, indicating that such infections are largely driven by eco-environmental conditions favouring the infestation of arthropod vectors that transmit pathogens to cats. To date, no studies have investigated the presence of arthropod vectors in cats from Cyprus, however ectoparasites described in Cyprus include the ticks *Rhipicephalus sanguineus*, *Rhipicephalus pusillus*, *Ixodes ventalloi* [2], the cat flea *Ctenocephalides felis* [5] and phlebotomine sand flies such as *Phlebotomus tobbi*, *Phlebotomus galilaeus*, and *Phlebotomus papatasi* [6].

## Conclusions

The results from this study demonstrate that FVBP, feline haemoplasmas and retroviral infections are present with considerable prevalence in the feline population of Cyprus. These findings should alert owners, the veterinary community and public health authorities to the possible risk of transmission of zoonotic FVBP including *B. henselae* and *L. infantum.* Priority should be given to establishing a surveillance system for arthropod vectors and FVBP in cats in order to monitor their distribution and prevent further spreading of these pathogens with regular effective prophylactic measures, such as the use of ectoparasite prevention in cats.

## Additional files

Additional file 1: Sequences of the four amplicons from the positive confirmatory *Leishmania* spp. qPCR. (TXT 971 bytes)
Additional file 2: Table S1.
*P-*values, *χ* ^2^ and degrees of freedom derived from Chi-square analysis for variables in relation to infectious agent or group of infectious agents. (DOCX 35 kb)
Additional file 3: Table S2.
*P*-and *Z*-values derived from Mann-Whitney U-tests for age in relation to infectious agent or group of infectious agents. (DOCX 16 kb)

## Abbreviations

CI: Confidence interval
CMhm: “*Candidatus* Mycoplasma haemominutum”
CMt: “*Candidatus* Mycoplasma turicensis”
Ct: Threshold cycle
FeLV: Feline leukaemia virus
FIV: Feline immunodeficiency virus
FVBP: Feline vector-borne pathogens
Mhf: *Mycoplasma haemofelis*
OR: Odds ratio
qPCR: Quantitative polymerase chain reaction
VBP: Vector-b7orne pathogens
