# Supplementary material for: Prevalence study and risk factor analysis of selected bacterial, protozoal and viral, including vector-borne, pathogens in cats from Cyprus
Source: Parasit Vectors. 2017 Mar 13;10:130. doi: 10.1186/s13071-017-2063-2 (PMC5346881; doi:10.1186/s13071-017-2063-2)
Supplement: Additional file 2: Table S1. — P-values, χ 2 and degrees of freedom derived from Chi-square analysis for variables in relation to infectious agent or group of infectious agents. (DOCX 35 kb) [file 13071_2017_2063_MOESM2_ESM.docx]

| **Additional file 2. Table S1** *P-*values, χ^2^ and degrees of freedom derived from Chi-square analysis for variables in relation to infectious agent or group of infectious agents. *P-*values < 0.2 but > 0.05 are shown in italics. Significant *P-*values ≤ 0.05 are shown in bold | | | | | | | | | | | | | |
| --- | --- | --- | --- | --- | --- | --- | --- | --- | --- | --- | --- | --- | --- |
| **Variable** | **Mhf PCR**  **Positive**  ***P***  **χ^2^, df** | **CMhm PCR**  **Positive**  ***P***  **χ^2^, df** | **CMt PCR**  **Positive**  ***P***  **χ^2^, df** | **Any hp PCR positive**  ***P***  **χ^2^, df** | ***B. henselae* PCR**  **Positive**  ***P***  **χ^2^, df** | ***L. infantum* PCR**  **Positive**  ***P***  **χ^2^, df** | ***L. infantum* serology**  **Positive**  ***P***  **χ^2^, df** | ***L. infantum* infection positive**  ***P***  **χ^2^, df** | **FeLV serology**  **Positive**  ***P***  **χ^2^, df** | **FIV serology**  **Positive**  ***P***  **χ^2^, df** | **Retroviral serology**  **Positive**  ***P***  **χ^2^, df** | ***Hepatozoon* spp. PCR**  **Positive**  ***P***  **χ^2^, df** | **FVBP**  **Positive**  ***P***  **χ^2^, df** |
| **Gender**  Male  Female (Ref.) | 0.920  0.010, 1 | 0.668  0.183, 1 | 0.709  0.139, 1 | 0.896  0.017, 1 | 0.813  0.056, 1 | 0.833  0.044, 1 | 0.854  0.034, 1 | 0.682  0.168, 1 | 0.202  1.144, 1 | 0.585  0.338, 1 | *0.173*  1.663, 1 | 0.448  0.575, 1 | 0.420  0.627, 1 |
| **Breed**  Non-Pedigree  Pedigree (Ref.) | 0.250  1.325, 1 | *0.161*  1.967, 1 | 0.270  1.216, 1 | *0.069*  3.299, 1 | *0.156*  2.012, 1 | 0.534  0.386, 1 | 0.424  0.647, 1 | 0.331  0.943, 1 | 0.270  1.072, 1 | *0.051*  3.880, 1 | **0.020**  5.326, 1 | **0.009**  6.815, 1 | **0.020**  9.936, 1 |
| **Housing**  Access to outdoors  Indoors only (Ref.) | **0.041**  1.194, 1 | **0.001**  10.473, 1 | 0.590  0.291, 1 | **0.001**  12.273, 1 | *0.171*  1.871, 1 | 0.269  1.222, 1 | *0.148*  2.125, 1 | *0.078*  3.097, 1 | *0.075*  2.039, 1 | **0.015**  6.087, 1 | 0.263  1.151, 1 | *0.121*  2.400, 1 | *0.062*  3.488, 1 |
| **Lifestyle**  Shelter-feral  Owned (Ref.) | *0.086*  2.943, 1 | **0.026**  4.936, 1 | **0.007**  7.164, 1 | **0.042**  4.144, 1 | *0.187*  1.744, 1 | 0.597  0.061, 1 | *0.146*  2.210, 1 | *0.118*  2.446, 1 | 0.335  0.679, 1 | **0.001**  14.718, 1 | **0.012**  7.286, 1 | **0.009**  6.869, 1 | **0.021**  5.385, 1 |
| **Habitat**  Rural  Urban (Ref.) | 0.201  1.633, 1 | *0.169*  1.888, 1 | 0.348  0.880, 1 | *0.175*  1,839, 1 | 0.650  0.206, 1 | 0.515  0.280, 1 | 0.799  0.052, 1 | 0.933  0.007, 1 | 0.526  0.199, 1 | 0.474  0.432, 1 | 0.946  0.019, 1 | **0.007**  7.264, 1 | **0.003**  8.920, 1 |
| **District** | 0.370  5.388, 5 | *0.195*  7.361, 5 | *0.136*  8.383, 5 | **0.017**  13.769, 5 | 0.416  4.995, 5 | 0.205  7.980, 5 | *0.140*  8.240, 5 | 0.414  5.015, 5 | *0.105*  2.456, 5 | 0.533  4.121, 5 | 0.341  5.749, 5 | 0.728  2.816, 5 | 0.843  2.031, 5 |
| **Travel history**  Never travelled abroad Travelled abroad (Ref.) | 0.233  1.325, 1 | 0.655  0.357, 1 | 0.915  0.001, 1 | 0.891  0.000, 1 | 0.832  0.098, 1 | 0.534  0.386, 1 | 0.371  0.757, 1 | 0.294  1.103, 1 | 0.262  1.072, 1 | 0.503  0.627, 1 | 0.972  0.046, 1 | **0.028**  4.815, 1 | **0.025**  6.010, 1 |
| **Health status**  Non-healthy  Healthy (Ref.) | *0.139*  2.188, 1 | **0.011**  6.545, 1 | 0.981  0.001, 1 | **0.011**  6.441, 1 | 0.864  0.029, 1 | 0.246  1.344, 1 | 0.453  0.590, 1 | 0.214  1.546, 1 | **0.049**  3.550, 1 | **0.010**  6.764, 1 | **0.003**  8.641, 1 | **0.003**  9.061, 1 | **0.008**  7.052, 1 |
| **Vaccination status**  Never vaccinated  Vaccinated (Ref.) | **0.006**  7.568, 1 | 0.960  0.003, 1 | **0.017**  5.660, 1 | 0.420  0.650, 1 | 0.445  0.582, 1 | 0.876  0.024, 1 | **0.036**  4.474, 1 | *0.063*  3.445, 1 | 0.514  0.628, 1 | 0.943  0.012, 1 | 0.861  0.114, 1 | **0.006**  7.422, 1 | **0.006**  7.547, 1 |
| **Ectoparasitic**  **prevention status**  Never used  Used (Ref.) | 0.634  3.455, 1 | 0.226  1.464, 1 | *0.123*  2.377, 1 | 0.054  3,708, 1 | 0.349  0.878, 1 | 0.603  0.270, 1 | *0.149*  2.109, 1 | *0.072*  3.230, 1 | *0.186*  1.120, 1 | **0.044**  4.147, 1 | 0.429  0.940, 1 | **0.007**  7.237, 1 | **0.008**  7.014, 1 |
| **Anaemia**  Anaemic  Non-anaemic (Ref.) | 0.344  0.728, 1 | *0.110*  2.557, 1 | 0.274  1.198, 1 | 0.334  0.934, 1 | 0.482  0.495, 1 | 0.882  0.022, 1 | 0.087  3.230, 1 | *0.133*  2.511, 1 | **0.006**  6.456, 1 | **0.025**  5.972, 1 | **0.002**  8.777, 1 | 0.202  1.630, 1 | 0.876  0.041, 1 |
| **Mhf PCR status**  Positive  Negative (Ref.) | na | na | na | na | 0.596  0.288, 1 | *0.177*  1.820, 1 | 0.369  0.807, 1 | *0.064*  3.446, 1 | 0.682  0.185, 1 | *0.129*  2.304, 1 | 0.338  0.917, 1 | 0.219  1.512, 1 | 0.073  3.218, 1 |
| **CMhm PCR status**  Positive  Negative (Ref.) | na | na | na | na | 0.962  0.002, 1 | *0.143*  2.143, 1 | *0.137*  2.210, 1 | 0.201  1.280, 1 | 0.421  0.646, 1 | **0.001**  28.371, 1 | **0.001**  20.371, 1 | *0.094*  2.808, 1 | *0.169*  1.888, 1 |
| **CMt PCR status**  Positive  Negative (Ref.) | na | na | na | na | 0.768  0.089, 1 | *0.148*  2.090, 1 | **0.013**  6.225, 1 | **0.001**  16.240, 1 | 0.744  0.108, 1 | **0.001**  15.247, 1 | **0.002**  9.848, 1 | **0.006**  7.523, 1 | **0.001**  11.129, 1 |
| **Any Haemoplasma PCR status**  Positive  Negative (Ref.) | na | na | na | na | 0.990  0.000, 1 | 0.280  1.169, 1 | 0.510  3.815, 1 | **0.044**  4.044, 1 | 0.743  0.108, 1 | **0.001**  25.255, 1 | **0.001**  16.519, 1 | *0.107*  2.601, 1 | **0.035**  4.458, 1 |
| ***L. infantum* infection status**  Positive  Negative (Ref.) | *0.064*  3.442, 1 | 0.201  1.280, 1 | **0.001**  16.240, 1 | **0.044**  4.044, 1 | 0.231  1.441, 1 | na | na | na | 0.770  0.076, 1 | *0.152*  2.008, 1 | 0.216  1.530, 1 | **0.001**  10.501, 1 | na |
| ***B*. *henselae* PCR status**  Positive  Negative (Ref.) | 0.596  0.288, 1 | 0.962  0.002, 1 | 0.768  0.089, 1 | 0.990  0.000, 1 | na | 0.479  0.502, 1 | 0.367  0.813, 1 | 0.231  1.441, 1 | 0.252  1.313, 1 | 0.315  1.008, 1 | 0.732  0.126, 1 | 0.691  0.158, 1 | na |
| **Retroviral serology status**  Positive  Negative (Ref.) | 0.338  0.917, 1 | **0.001**  20.371, 1 | **0.002**  9.848, 1 | **0.001**  16.519, 1 | 0.732  0.126, 1 | 0.977  0.001, 1 | 0.234  1.454, 1 | 0.216  1.530, 1 | na | na | na | **0.045**  4.037, 1 | 0.202  1.595, 1 |
| ***Hepatozoon* spp. PCR status**  Positive  Negative (Ref.) | 0.219  1.512, 1 | *0.094*  2.808, 1 | **0.006**  7.523, 1 | *0.107*  2.601, 1 | 0.691  0.158, 1 | **0.010**  6.699, 1 | **0.010**  6.585, 1 | **0.001**  10.501, 1 | 0.461  0.539, 1 | **0.048**  3.894, 1 | **0.045**  4.037, 1 | na | na |

*Abbreviations*: *Mhf* *Mycoplasma haemofelis*, *df* degrees of freedom, *CMhm* "*Candidatus* Mycoplasma haemominutum", *CMt* "*Candidatus* Mycoplasma turicensis", *Any hp* positivity in at least one of the following haemoplasma PCRs; Mhf, CMhm and CMt, *B. henselae Bartonella henselae*, *L. infantum Leishmania infantum* confirmed by DNA sequencing following confirmatory quantitative PCR, *L. infantum infection* positive DNA sequencing for *L*. *infantum* following confirmatory qPCR and/or positive *L*. *infantum* ELISA, *FeLV* feline leukaemia virus, *FIV* feline immunodeficiency virus, *Retroviral serology* positive for FeLV and/or FIV serology, *FVBP* positive for at least one of the PCRs for *B. henselae*, *Ehrlichia/Anaplasma* spp. and/or *Hepatozoon* spp., and/or *L*. *infantum* infection (i.e. positive DNA sequencing for *L*. *infantum* following confirmatory qPCR and/or positive *L*. *infantum* ELISA), *Ref.* reference category, *na* not applicable
