# Supplementary material for: Prevalence study and risk factor analysis of selected bacterial, protozoal and viral, including vector-borne, pathogens in cats from Cyprus
Source: Parasit Vectors. 2017 Mar 13;10:130. doi: 10.1186/s13071-017-2063-2 (PMC5346881; doi:10.1186/s13071-017-2063-2)
Supplement: Additional file 3: Table S2. — P-and Z-values derived from Mann-Whitney U-tests for age in relation to infectious agent or group of infectious agents. (DOCX 16 kb) [file 13071_2017_2063_MOESM3_ESM.docx]

**Additional file 3. Table S2** *P*-and *Z*-values derived from Mann-Whitney U-tests for age in relation to infectious agent or group of infectious agents. *P-*values < 0.2 but > 0.05 are shown in italics. Significant *P-*values ≤ 0.05 are shown in bold

|  | **Mhf PCR**  **positive** | **CMhm PCR**  **positive** | **CMt PCR**  **positive** | **Any hp PCR positive** | ***B. henselae* PCR**  **positive** | ***L. infantum* PCR**  **positive** | ***L. infantum* serology**  **positive** | ***L. infantum* infection**  **positive** | **FeLV serology**  **positive** | **FIV serology**  **positive** | **Retroviral serology**  **positive** | ***Hepatozoon* spp. PCR**  **positive** | **FVBP**  **positive** |
| --- | --- | --- | --- | --- | --- | --- | --- | --- | --- | --- | --- | --- | --- |
| **P** | 0.934 | **0.014** | 0.584 | *0.055* | *0.152* | 0.712 | 0.849 | 0.560 | **0.023** | 0.288 | 0.760 | 0.937 | 0.530 |
| **Z** | -0.083 | -2.445 | -0.548 | -1.919 | -1.434 | -0.735 | -0.171 | -0.558 | -2.132 | -1.019 | -0.376 | -0.081 | -0.528 |

*Abbreviations*: *Mhf* *Mycoplasma haemofelis*, *CMhm* "*Candidatus* Mycoplasma haemominutum", *CMt* "*Candidatus* Mycoplasma turicensis", *Any hp* positivity in at least one of the following haemoplasma PCRs; Mhf, CMhm and CMt, *B. henselae Bartonella henselae*, *L. infantum Leishmania infantum* confirmed by DNA sequencing following confirmatory quantitative PCR, *L. infantum infection* positive DNA sequencing for *L*. *infantum* following confirmatory qPCR and/or positive *L*. *infantum* ELISA, *FeLV* feline leukaemia virus, *FIV* feline immunodeficiency virus, *Retroviral serology* positive for FeLV and/or FIV serology, *FVBP* positive for at least one of the PCRs for *B. henselae*, *Ehrlichia/Anaplasma* spp. and/or *Hepatozoon* spp., and/or *L*. *infantum* infection (i.e. positive DNA sequencing for *L*. *infantum* following confirmatory qPCR and/or positive *L*. *infantum* ELISA)
